# Catalytic methyl esterification of colophony over ZnO/SFCCR with subcritical CO_2_: catalytic performance, reaction pathway and kinetics

**DOI:** 10.1098/rsos.172124

**Published:** 2018-05-02

**Authors:** Xubin Wang, Linlin Wang, Xiaopeng Chen, Dan Zhou, Han Xiao, Xiaojie Wei, Jiezhen Liang

**Affiliations:** 1 Guangxi Key Laboratory of Petrochemical Resource Processing and Process Intensification Technology, School of Chemistry and Chemical Engineering, Guangxi University, Nanning 530004, People's Republic of China; 2 College of Chemistry and Molecular Sciences, Wuhan University, Wuhan 430072, People's Republic of China

**Keywords:** colophony, subcritical CO_2_, methyl esters, reaction pathway, kinetics

## Abstract

A heterogeneous catalyst (ZnO/SFCCR) composed of ZnO supported on spent fluid cracking catalyst by wet impregnation was synthesized and applied to the esterification of colophony acids with methanol under subcritical CO_2_ conditions. The catalyst was characterized by SEM-EDS, BET, ICP, FTIR, XRD and Py-IR. An experimental set-up involving a new injection technique was designed to promote the heterogeneous methyl esterification, and the subcritical CO_2_ played a role in auxiliary acid catalysis (a pH range of 3.54–3.91), increasing the lifespan of ZnO/SFCCR, reducing the viscosity of the system to promote gas–liquid mass transfer. A maximum conversion rate of 97.01% was obtained in a relatively short time of 5 h. Kinetic experiments were performed from 190 to 220°C using a special high-temperature sampling device and analysing aliquots with high-performance liquid chromatography. A new reaction pathway, involving methyl abietate, methyl dehydroabietate, methyl neoabietate and methyl palustrate along with other kinds of colophony acids, was developed. The kinetic parameters were obtained using the Levenberg–Marquardt nonlinear least-squares method, and the activation energies for the isomerizations of neoabietic and palustric acids and for the methyl esterification of neoabietic, abietic, palustric and dehydroabietic acids were found to be 107.09, 113.95, 68.99, 49.85, 75.43 and 59.20 kJ mol^−1^, respectively. The results from the kinetic model were in good agreement with experimental values.

*Highlights*

— Using ZnO/SFCCR with subcritical CO_2_ as catalyst-synthesized CMEs.
— The conversion of CMEs reached 97.01% in 5 h.
— A new reaction scheme together with isomerization and methyl esterification kinetic model.

## 1. Introduction

There has been a wealth of interdependent research focused on creating high-value products from renewable biomass as fossil fuel resources deplete [1]. Colophony is a natural resin obtained by distilling pine resin, which comprises greater than 90% resin acids with a single carboxylic group [2,3]. Colophony can be modified by changing the carboxyl group structure to broaden the range of applications and increase its value [4,5]. Esterification is one of the most common modification techniques because this process reduces the acid value, improves thermal stability and increases acid and alkali resistance. Colophony methyl esters (CMEs) are widely used in the adhesives, coatings and food industries, and act as solvents and plasticizers and as important functional components in varnishes [6,7].

Fluid catalytic cracking is an important process in petroleum refining, which generates large quantities of spent fluid cracking catalyst (SFCCR)—approximately 160 000 tons [8]. Spent catalysts that are not regenerated are typically buried and not only pollute the environment but also limit recycling of silicon and aluminium resources [9]. SFCCR has been reported as a material for use in sorbent and construction applications; however, SFCCR has rarely been employed as a catalyst [10,11]. In fact, as SFCCR is composed primarily of SiO_2_ and Al_2_O_3_ and has a stable molecular sieve structure with a large specific surface area, it may find use as a suitable catalytic support.

The esterification reaction typically proceeds under harsh reaction conditions, e.g. high temperatures and long reaction times, in the presence of a catalyst as a result of the steric effects of the colophony acid tricyclic skeleton structure [8]. Conventional alkali and protonic acid catalysts were soon abandoned because of the sensitivity of the colophony chromophores and as a result of serious reactor corrosion [12–14]. Solid acid catalysts such as ZnO are widely used in colophony esterification because of their high catalytic activity, non-corrosive nature to reactors and equipment, and the low dosage required. However, the primary challenge associated with homogeneous catalysts is how to separate the catalyst from the product, which can lead to the presence of residual metals in the product [15,16]. Therefore, the development of new heterogeneous catalysts is of significant interest [9,17].

Recently, there have been a growing number of studies concerning the use of sub/supercritical CO_2_ as an inexpensive, non-toxic, non-flammable and renewable alternative [18–20]. Sub/supercritical CO_2_ can be combined with water generated from a high-temperature compression system to produce carbonic acid. CO_2_ can effectively adjust the amount of water and the pH, act as an auxiliary catalyst and enhance mass transfer during a reaction [21,22]. Additionally, numerous reports have identified that supercritical CO_2_ can improve the useable lifespan of a catalyst by decreasing coke formation [18]. Sub/supercritical CO_2_ has been accepted as a green catalytic medium with potential applications in organic reactions.

Traditionally, CMEs are prepared using the homogeneous catalyst ZnO in conjunction with high temperatures and prolonged reaction times [16]. However, there are no reports relating to methyl esterification reactions catalysed by heterogeneous catalysts that promote mass transfer. Furthermore, no specific reports have been published concerning the reaction pathways and kinetics of colophony acid esterification with methanol. In this work, a novel experimental set-up was designed to increase the gas–liquid mass transfer, and the methyl esterification of colophony over ZnO/SFCCR in the presence of subcritical CO_2_ (sub-CO_2_) was explored, including the reaction pathway and kinetics.

## 2. Experimental procedure

### 2.1. Materials

Technical grade colophony (acid value 175.76 mg KOH/g) was supplied by Guangxi Wuzhou Pine Chemicals Ltd, China. Methanol (analytical grade), potassium hydroxide (KOH, analytical grade), zinc chloride (ZnCl_2_, analytical grade) and methanol (chromatographic grade) were purchased from the Guangdong Guanghua Sci-Tech Co., Ltd, China. SFCCR was obtained from the Petro China Guangxi Tiandong Petrochemical Co., Ltd, China. Carbon dioxide (99.9%) and nitrogen (greater than 99.0%) were supplied by Nanning Air Separation Gas Co., Ltd, China.

### 2.2. Catalyst preparation and characterization

The ZnO was loaded onto the SFCCR using a wet impregnation method. A quantity of SFCCR was placed in a muffle furnace and calcined at 500°C for 3 h to remove coke deposited on the surface and in the pores, after which 39 g of the calcined material was combined with 21.8 g of ZnCl_2_ along with an amount of deionized water. This was accomplished by first dissolving the ZnCl_2_ in the deionized water and pouring the solution over the SFCCR, followed by vigorous stirring with a glass rod. The mixture was subsequently placed in a vacuum oven and kept overnight at a pressure of 0.04 MPa. Finally, the ZnO/SFCCR was dried at 110°C for 6 h and then calcined at 450°C for 4 h under atmospheric pressure.

The surface morphology of the resulting catalyst was investigated by scanning electron microscopy (SEM) with a Hitachi SU-8220 having an energy-dispersive spectroscopy (EDS) attachment. The specific surface area was determined using the BET method in conjunction with a Gemini VII 2390 instrument. The actual loading of Zn was determined by inductively coupled plasma–atomic emission spectroscopy (ICP-AES) with a Perkin Elemer Optima 8000. The X-ray powder diffraction (XRD) patterns of the catalyst were obtained using a Rigaku Smartlab X-ray diffractometer. Scans were from 10° to 90° (2*θ*) at a rate of 6° min^−1^ with a step size of 0.02°. Pyridine adsorption-IR spectroscopy (Py-IR) data were acquired at 150 and 350°C with a Perkin-Elmer 2000 FTIR spectrometer.

### 2.3. Experimental set-up and procedure

**Figure 1.**
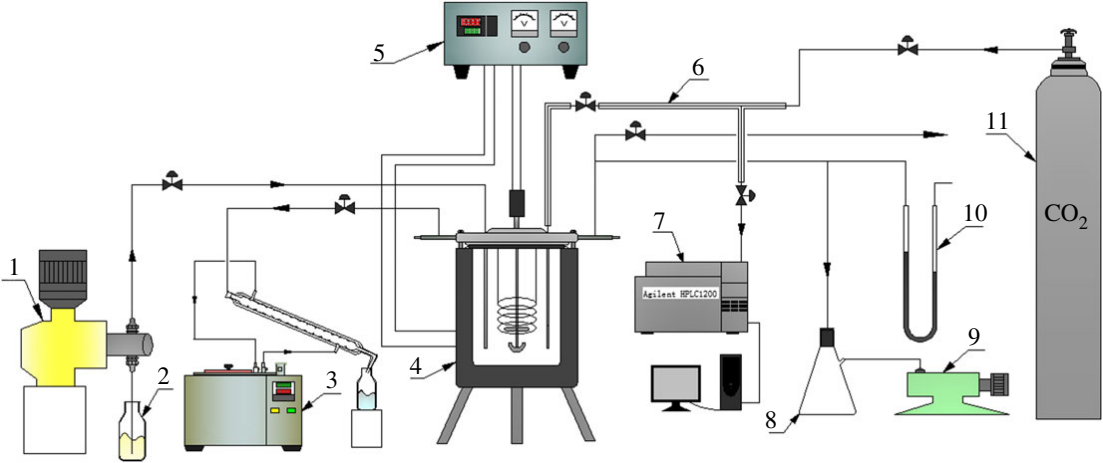
The experimental set-up. Legend: (1) plunger pump, (2) methanol reservoir, (3) constant temperature water-bath, (4) autoclave, (5) power controller, (6) sampling pipe, (7) high-performance liquid chromatography, (8) vacuum flask, (9) vacuum pump, (10) mercury U-tube manometer and (11) CO_2_ cylinders.

Reactions were carried out in a 2 l autoclave made of 316 stainless steel (Dalian Jingyi Autoclave Co., Ltd, China) with a maximum pressure of 21.5 MPa. The temperature and pressure in the reactor were measured using an EA-2 thermocouple (Dalian Jingyi Autoclave Co., Ltd, China) and a precision pressure gauge (Xi'an Automation Instrument Manufacturing Co., Ltd, China). Methanol was pumped into the reactor with a J-SX2/50 plunger pump (Zhejiang Ligao Pump Technology Co., Ltd, China). As noted, to enhance the gas–liquid mass transfer, the methanol injection tube was designed to extend to the bottom of the reactor to increase the residence time of methanol in the liquid phase. Moving the injection tube from the top of the autoclave into the liquid phase was found to change the conversion rate under the same conditions (table 1). A special sampling device was also designed that extended to the base of the reactor. This pipe was wrapped with a laboratory-made ribbon heater to prevent solidification of products and thus keep the pipe from being blocked. The end of the sampling pipe was equipped with a 10 µm filter to prevent the removal of the catalyst. A schematic of the reaction system is shown in figure 1.

**Table 1.** The conversion rate of different injection position.

reaction time (h)	1	2	3
conversions (injection tube to the base, %)	56.83	73.65	84.91
conversions (injection tube to the top, %)	49.87	61.25	70.03

Prior to each trial, 600 g of colophony was crushed to a particle size of less than 15 mm and then transferred into the autoclave to be heated. When the temperature exceeded 120°C, the catalyst was added (at 1 wt%) to the autoclave and the heating device was turned off. The reactor was then closed and sealed. Following this, the air in the autoclave was pumped out to obtain an absolute pressure of approximately 0.008 MPa, followed by charging with 2 MPa N_2_ for 10 min to leak-check the system. N_2_ was subsequently removed and the reactor refilled with N_2_ to 0.5 MPa. This was repeated three times to remove any remaining oxygen. The heating device was restarted and the reaction mixture was stirred at 100 r.p.m. When the temperature reached 200°C, methanol was pumped into the reactor and CO_2_ was added at the desired pressure, after which the reactor temperature was controlled at the desired value and the stirring speed was increased to 400 r.p.m. To maintain the reaction in the forward direction, the steam generated in the autoclave (consisting of water and methanol vapour) was discharged at the same intervals. The refractive index of the reaction mixture was measured with an Abbe refractometer to calculate the methanol concentration, then the methanol and CO_2_ were replenished as needed. Finally, when the reaction was complete, the heating device was stopped and the reactor was cooled to 150°C, after which the catalyst was separated from products to obtain high-quality CMEs. The separated catalysts were washed with turpentine and dried at 100°C for 5 h for recycling.

### 2.4. Sample withdrawal and analysis

Variations in the concentrations of reactants and products over time were assessed by withdrawing aliquots from the reaction system using the high-temperature sampling device. During the process, a valve on the device was slowly opened and a sample volume of approximately 1 cm^3^ was withdrawn every 5 min during the first 20 min, every 10 min to 60 min, every 15 min to 120 min, every 20 min to 180 min, and then every 30 min until the end of the reaction. Then the pipe was flushed with CO_2_ to clean out any residual sample in preparation for the next sampling.

The reaction species in the samples were quantitatively analysed by HPLC with an Agilent-1200 HPLC equipped with a reverse-phase 5 µm LC-18 column (250 × 4.6 mm, Agilent Technologies, USA). Chromatography grade methanol and ultra-pure water were used as the mobile phase, and operating conditions were as follows: sample concentration of 0.42% w/v, UV detection at 241 nm, methanol/water mobile phase 90 : 10 (v : v), flow rate of 1.5 ml min^−1^ and injection volume of 20 µl.

The reaction products were also qualitatively analysed by liquid chromatography with tandem mass spectrometry (LC-MS/MS) using a TripleTOF 5600+ system. The MS parameters were as follows: capillary voltage of 3.0 kV, electrospray ionization source block and desolvation temperatures of 110 and 350°C; cone nitrogen and desolvation gas flows of 50 and 650 l h^−1^, respectively.

The acid values and viscosities of products were used to determine the quality of the CMEs [23]. Acid values were converted to conversion rates using the following equation:

2.1 $$\text{Conversion}=\frac{V_{C}-V_{E}}{V_{C}}\times 100\%,$$

where *V*_C_ represents the colophony acid value and *V*_E_ represents the acid value of the CMEs, both determined as outlined in the ASTM D465-82 standard. Besides, viscosity is another parameter that evaluates CME, as this factor determines the suitability of the material for applications such as adhesives, coatings and soldering flux. For such uses, the viscosity should be between 3000 and 6600 mPa s at 25°C based on the ASTM D1824-66 standard.

## 3. Results and discussions

### 3.1. Catalyst characterization

**Figure 2.**
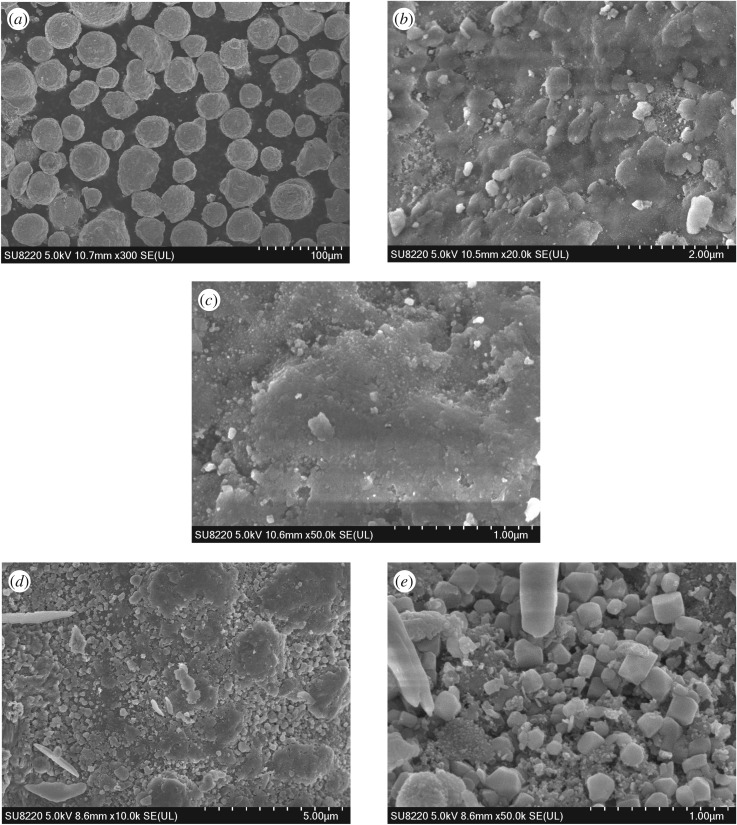
SEM micrograph of the samples : SFCCR (*a,b*), activated SFCCR (*c*), ZnO/SFCCR (*d,e*).

The surface morphologies of the SFCCR and ZnO/SFCCR were analysed by SEM and the surface elemental compositions were determined by EDS, with the results shown in figure 2 and electronic supplementary material, table S1. As shown in figure 2*a*, SFCCR is porous, coarse and spherical, which identifies the large BET surface area. Besides, the as-received SFCCR particles were also clogged with internal coke deposits, as indicated by the microphotograph in figure 2*b*. After calcination at high temperature (figure 2*c*), it can be seen that the coke deposits were removed a lot and pores are evident. In the case of the ZnO/SFCCR catalyst (figure 2*d,e*), large quantities of ZnO crystallites with a particular cuboid structure are observed in the pores of SFCCR carriers.

The EDS results for the ZnO/SFCCR (electronic supplementary material, table S1) demonstrate that oxygen was present in the highest proportion, followed by Zn, Al and Si. The Zn loading of 17.94% is in good agreement with the ICP results and the theoretical value, as discussed further on in this section.

The specific surface areas of the SFCCR and ZnO/SFCCR were determined and are provided in electronic supplementary material, table S2. The micropore volumes and pore diameters were quite small, and the surface area of the SFCCR following heating is seen to have increased to some extent. This result is attributed to removal of carbon deposits on the surface by the high-temperature calcination. However, the SFCCR itself is deactivated and the pore structure has collapsed and is non-renewable, so the specific surface area, pore size and capacity have not increased substantially. After loading, the ZnO/SFCCR has a larger specific surface area and greater pore size, due to the highly dispersed ZnO particles inside the catalyst. This result is consistent with the SEM analysis.

ICP-AES was used to examine the compositions of the catalysts, especially the actual loading of ZnO in the ZnO/SFCCR compared with the theoretical loading of 25%. The results are shown in electronic supplementary material, table S3. The SFCCR was primarily composed of Al_2_O_3_ and SiO_2_ and, as expected, no ZnO was detected in this material. The ZnO loading on the ZnO/SFCCR was 20.85%. This value is only slightly lower than the theoretical value, indicating that the wet impregnation method is a suitable means of preparing the heterogeneous catalyst.

**Figure 3.**
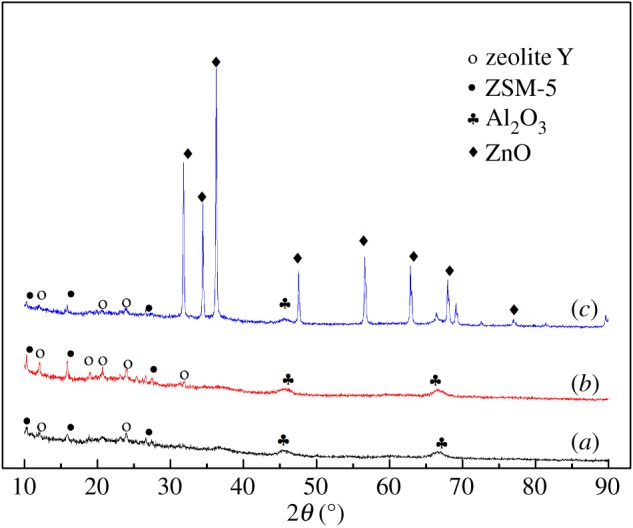
XRD patterns of SFCCR (*a*), SFCCR after calcination (*b*) and ZnO/SFCCR (*c*).

Figure 3 displays the XRD patterns of the SFCCR before and after calcination, and of the ZnO/SFCCR. The SFCCR contains different crystalline zeolite phases, including Y zeolite and ZSM-5 zeolite and Al_2_O_3_ that are observed through XRD. The diffraction peaks of Y zeolite are enhanced with a higher crystallinity in activated SFCCR, which is caused by the removal of cracking residues on the surface. Compared with SFCCR, the ZnO/SFCCR shows a group of diffraction peaks consistent with the standard of ZnO wurtzite. The diffraction peaks at 32.2°, 34.7°, 36.5°, 47.7°, 56.8°, 63.0° and 77.1° can be ascribed respectively to the ZnO crystal planes (JCPDS No. 36-1451). These data demonstrate that a significant quantity of ZnO crystallites was loaded into the SFCCR carrier, in agreement with the SEM analysis.

The FTIR spectra of SFCCR and ZnO/SFCCR are presented in electronic supplementary material, figure S1. The bands at 1090 and 452 cm^−1^ in the SFCCR spectrum (electronic supplementary material, figure S1a) are assigned to the stretching and bending vibrations of Si-O-T bonds (T: tetrahedral Si or Al). The bands at 2934 and 828 cm^−1^ correspond to C–H stretching and bending vibrations, due to the presence of surface coke on the SFCCR and ZnO/SFCCR, in agreement with the SEM results. The bands at 3520 and 1640 cm^−1^ are attributed to the bending vibrations of physically absorbed water. The strong absorption peak generated at 472 cm^−1^ by the ZnO/SFCCR is a characteristic of Zn–O bonds.

The Py-IR spectra of SFCCR and ZnO/SFCCR are depicted in electronic supplementary material, figure S2. The peak at 1440 cm^−1^ is assigned to pyridine on a Lewis acid, while the peak at 1540 cm^−1^ corresponds to pyridine on a Bronsted acid. These results suggest that both the Lewis and Bronsted acidities of the ZnO/SFCCR were greater than those of the SFCCR and the bare ZnO, which tend to greatly promote the esterification reaction.

### 3.2. The acid-catalysed reaction

**Figure 4.**
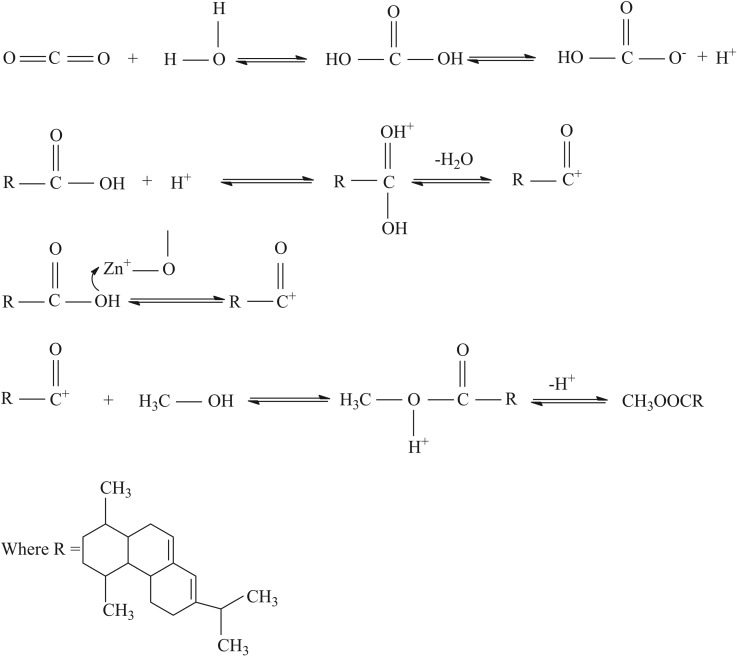
The mechanism of acid-catalysed methyl esterification.

The activity of the ZnO/SFCCR and the efficacy of sub-CO_2_ were assessed by performing trials using CO_2_, Al_2_O_3_ or ZnO alone and by a blank experiment with no catalyst at 220°C for 4 h. The results are presented in table 2. The ZnO exhibits higher catalytic activity than other solid acid catalysts like Al_2_O_3_, and thus improves the conversion rate. However, the ZnO/SFCCR shows superior catalytic activity due to its high specific surface area and smaller particle sizes. Under the same conditions, the conversion rate can be increased from 72.42 to 83.28%. CO_2_ is also able to promote this reaction by acting as a Lewis acid. When CO_2_ was added along with the ZnO/SFCCR, the conversion rate was as high as 94.08% based on auxiliary catalysis by the sub-CO_2_. In high-temperature compressed water systems, CO_2_ dissolves to form carbonic acid and so lowers the pH of the mixture, which in turn promotes the esterification reaction. In the present case, the pH was 3.66 at 220°C and 3.5 MPa. Santos *et al*. reported the acid catalysis effect of CO_2_ on esterification and Benazzi *et al*. confirmed that CO_2_ promotes hydrogenation reactions [24,25]. The mechanism of the acid-catalysed reaction is assumed to be as shown in figure 4.

**Table 2.** Effects of different catalysts on colophony methyl esterification.

the species of catalysts	dosages	conversion (%)
no catalyst		55.42
CO_2_	3.5 MPa	66.38
Al_2_O_3_	1.5 g	65.52
ZnO	1.5 g	72.42
ZnO/SFCCR	6.0 g	83.28
ZnO/SFCCR + CO_2_	6.0 g, 3.5 MPa	94.08

### 3.3. The effects of varying reaction factors

**Figure 5.**
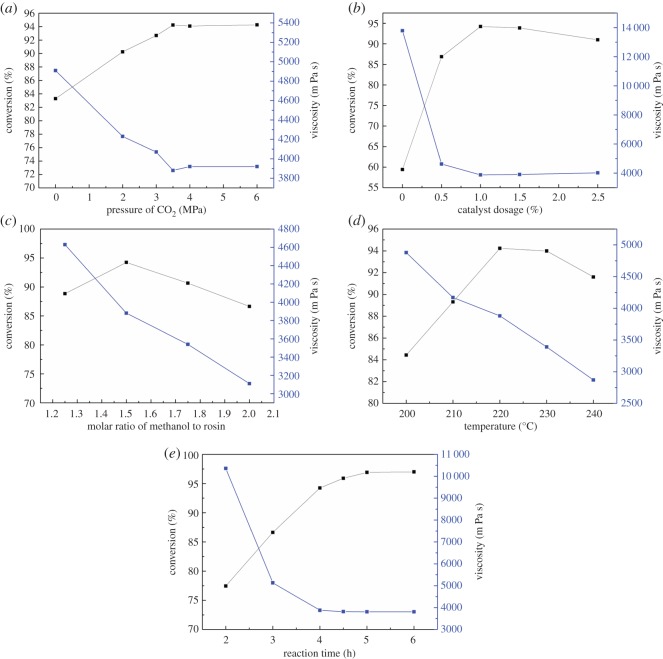
(*a*) The effects of the CO_2_ pressure on conversion and viscosity. Reaction conditions: catalyst amount = 1.0 wt%, methanol : colophony molar ratio = 1.5 : 1, temperature = 220°C, time = 4 h, agitator speed = 600 r.p.m. (*b*) The effects of the catalyst amount on conversion and viscosity. Reaction conditions: CO_2_ pressure = 3.5 MPa, methanol : colophony molar ratio = 1.5 : 1, temperature = 220°C, time = 4 h, agitator speed = 600 r.p.m. (*c*) The effects of the methanol : colophony molar ratio on conversion and viscosity. Reaction conditions: CO_2_ pressure = 3.5 MPa, catalyst amount = 1.0 wt%, temperature = 220°C, time = 4 h, agitator speed = 600 r.p.m. (*d*) The effects of reaction temperature on conversion and viscosity. Reaction conditions: CO_2_ pressure = 3.5 MPa, catalyst amount = 1.0 wt%, methanol : colophony molar ratio = 1.5 : 1, time = 4 h, agitator speed = 600 r.p.m. (*e*) The effects of reaction time on conversion and viscosity. Reaction conditions: CO_2_ pressure = 3.5 MPa, catalyst amount = 1.0 wt%, methanol : colophony molar ratio = 1.5:1, temperature = 220°C, agitator speed = 600 r.p.m.

The results of esterification reactions can be affected by a number of parameters, including the reaction temperature, the CO_2_ pressure, the catalyst amount, the molar ratio of colophony to methanol and the reaction time. The results of trials varying these factors are shown in figure 5.

The effects of CO_2_ pressure on the colophony esterification are summarized in figure 5*a*. A conversion rate of 83.28% was obtained without CO_2_ gas, indicating that some of the raw materials remained in the reactor without participating in the reaction. Compared with the ASTM standard value, this result is unsatisfactory. Using 2 MPa of CO_2_, the conversion rate was increased from 83.28 to 90.25%, demonstrating increased catalytic activity in the system. Increasing the pressure from 2 to 3.5 MPa also raised the conversion rate from 90.25 to 94.23%, confirming that the addition of sub-CO_2_ generates carbonic acid to adjust the pH while reducing the amount of water and thus promoting the progress of the reaction [19]. However, further increasing the pressure from 3.5 to 6 MPa had no significant effect, possibly as a result of the relationship between the pH of the CO_2_–H_2_O system and the pressure. The pH value in this system can be calculated by the following equation [26]:

3.1 $$\text{pH}=8.00\times 10^{-6}\times T^{2}+0.00209\times T-0.216\times \text{ln}(P_{\text{C}{\text{O}}_{2}})+3.92,$$

where *T* is the temperature in degrees Kelvin and $P_{\text{C}{\text{O}}_{2}}$ is the partial pressure of CO_2_ in the autoclave. The pH value of the system is predicted to be 3.91–3.66 at pressures from 2 to 3.5 MPa, and the pH values at 4 and 6 MPa are calculated to be 3.61 and 3.54, respectively. These data indicate that increases in the sub-CO_2_ pressure in this range have little effect on the reaction. In addition, operating at such pressures will increase the equipment costs, and so a CO_2_ pressure of 3.5 MPa is considered to be optimal.

The viscosity was evidently decreased with increases in the conversion rate. As the conversion increased from 83.28 to 90.25%, the viscosity of the CMEs decreased from 4910 to 4230 mPa s and finally stabilized around 3900 mPa s. This level of viscosity would permit the products to be used in most applications. Thus, again, the optimum CO_2_ pressure is approximately 3.5 MPa.

The other benefit of using sub-CO_2_ is that it can force the chemical equilibrium in the desired direction by removing water generated in the reaction system [20]. Besides, the addition of sub-CO_2_ can also affect the physical properties of the reaction system, such as reducing the viscosity to improve the gas–liquid mass transfer. The viscosity of colophony was calculated as 3.1313 mPa s by the Nash equation at 220°C, and the data were studied in another paper [27,28]. The viscosity of CMEs was measured as 1.2147 mPa s at 220°C. The viscosity of high-temperature system decreases with time, while adding sub-CO_2_ can further accelerate the trend of viscosity decline, thereby enhancing the gas–liquid mass transfer [22].

The effects of varying catalyst amounts on the conversion rate are graphed in figure 5*b*. Increasing the catalyst mass first decreased the acid value of the product, followed by a slight increase. The use of the catalyst greatly improved the efficiency of the esterification reaction to give products with low acid values. In the absence of a catalyst, the conversion rate was as low as 59.42% and the product viscosity was high. By contrast, adding 1.0 wt% of the catalyst raised the conversion rate to 94.23% and lowered the viscosity of the CMEs to 3880 mPa s, demonstrating the efficient activity of the supported catalyst. However, further increasing the catalyst loading resulted in a decrease in conversion, possibly caused by side reactions such as the oxidation of hydroxyl groups. Thus, the optimum catalyst : colophony mass ratio was determined to be 1.0 wt%.

Figure 5*c* shows the effects of the methanol : colophony molar ratio. The boiling point of methanol is low, meaning that the esterification reaction is actually a gas–liquid reaction and that methanol should be present in excess. Using a methanol : colophony molar ratio of 1.25 : 1, a conversion rate of 88.84% was obtained in conjunction with a high viscosity, indicating that much of the methanol remained as a separate phase in the upper reactor in the form of steam and so was not involved in the reaction. Increasing the molar ratio to 1.5 : 1 rapidly raised the conversion rate to 94.23% and lowered the viscosity to 3880 mPa s. Thus, this would seem to be the optimal methanol : colophony molar ratio. However, upon increasing the molar ratio to 2.0 : 1, a certain degree of rebound was observed such that the conversion decreased along with the viscosity, suggesting a side effect of the excess methanol. The viscosity is evidently sensitive to changes in the molar ratio, and a low viscosity value will reduce the quality of the CMEs.

The catalytic performance of ZnO/SFCCR during the colophony esterification at different temperatures under a CO_2_ pressure of 3.5 MPa for 4 h is summarized in figure 5*d*. The acid-catalysed esterification reaction has a high activation energy, and so the temperature requirements are very strict. As the temperature was increased from 200 to 220°C, the conversion rapidly rose from 84.43 to 94.23%, while further increasing the temperature to 240°C decreased the conversion to 91.60%. This is the result of a variety of factors. As the temperature increases, the viscosity of the reaction system decreases along with increases in the diffusion coefficient, due to increased mass transfer. Raising the temperature will also have some negative effects related to the nature of the esterification reaction. This is an exothermic reaction and so raising the temperature does not promote the reaction in the forward direction or reduce the pH of the system. As well, because of the catalytic cracking effects of the SFCCR, higher temperatures will lead to resin acid cracking and reduce the quality of the CME product.

The viscosity of CMEs is also affected by the reaction temperature. Increasing the temperature from 200 to 220°C caused the viscosity to decrease continuously from 4880 to 3880 mPa s, and then to further drop to 2870 mPa s at 240°C. The latter value is below 3000 mPa s and thus demonstrates the synthesis of a low-quality product at high temperature. Taking the conversion and viscosity into consideration, the remaining experiments were conducted at 220°C.

Figure 5*e* illustrates the effects of varying the reaction time on the conversion and viscosity of the products. Over the initial 4 h, the conversion rate increased rapidly as the viscosity decreased. Extending the reaction to 5 h, the conversion rate slowly increased to 96.92% and the viscosity stabilized at 3800 mPa s. At this point, the initial charge of colophony was almost exhausted and the reaction reached equilibrium. Further extending the reaction time not only had no effect on the conversion rate, but also darkened the product mixture and so reduced the quality of the CMEs.

In summary, the optimum reaction conditions for the ZnO/SFCCR catalysis of colophony methyl esterification are as follows: a CO_2_ pressure of 3.5 MPa, a catalyst : colophony mass ratio of 1.0%, a methanol : colophony molar ratio of 1.5 : 1, a reaction temperature of 220°C and a time of 5 h. Under these conditions, three verification experiments were carried out and the average conversion rate and viscosity were 97.01% and 3810 mPa s, respectively, with a high product yield of 89.43%, both of which are in accordance with the ASTM standard. Compared with the high temperature (more than 270°C) and long reaction time (7–11 h) that was reported in methyl esterification, this process has the advantage of improving productivity [29,30].

### 3.4. Assessing catalyst stability

Trials were subsequently performed to assess the stability of the catalyst and the extent to which sub-CO_2_ can effectively enhance the catalyst lifespan by reducing coke formation. Applying the optimum reaction conditions, CO_2_ and N_2_ were added in comparative experiments, and the catalyst was separated from products under a temperature of 150°C. The results of these trials are shown in electronic supplementary material, table S4. When sub-CO_2_ was not added, the catalyst exhibited good stability over the first three trials, after which it was gradually deactivated. By comparison, adding sub-CO_2_ improved the stability significantly, with only a slight decrease in the conversion rate after six runs. The decrease in the activity of catalysts is considered to be caused by the formation of carbonaceous coke on the catalyst surface [31]. Sub-CO_2_ displays higher solubilities than corresponding gases for heavy organics, which can accelerate the transfer of poisons from the internal and external catalyst surface and restrain coking, and thus verifies the stability of ZnO/SFCCR [32].

### 3.5. Reaction pathway and kinetic model

#### 3.5.1. The establishment of a kinetic model

**Figure 6.**
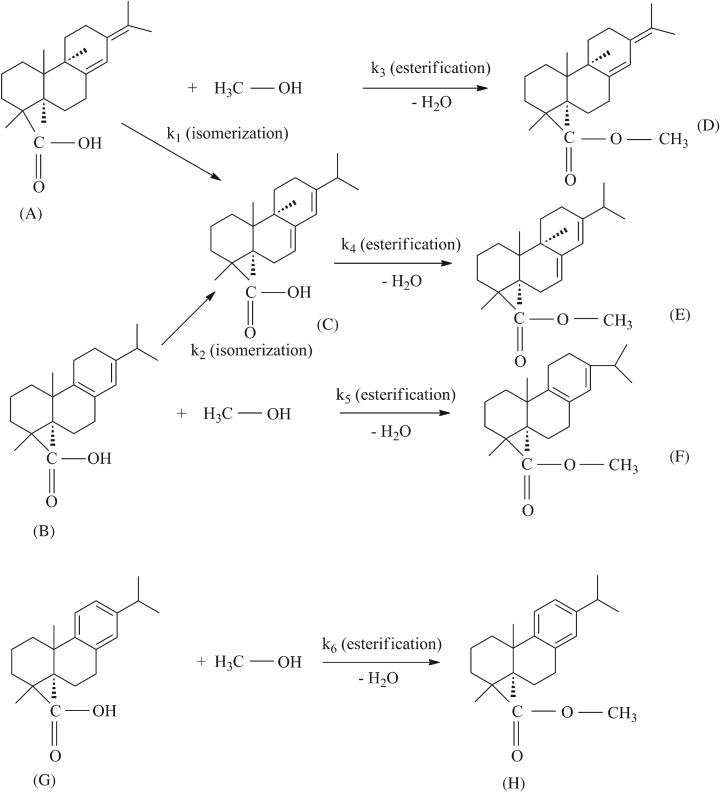
A proposed reaction pathway for the isomerization and esterification of colophony with methanol.

The composition of colophony is complex and consists of a variety of resin acids. The main components of the colophony used in this work were abietic acid (AA), palustric acids (PA), neoabietic acids (NA) and dehydroabietic acids (DA), and these compounds were able to undergo isomerization due to the presence of conjugated double bonds, while the CMEs include methyl abietate (MEA), methyl dehydroabietate (MEDA), methyl neoabietate (MENA) and methyl palustrate (MEPA) [33,34]. The catalysis of ZnO/SFCCR with sub-CO_2_ promoted the esterification reaction between the carboxyl groups of these acids and the methanol hydroxyl groups, but there have been no reports regarding the methyl esterification reactions of colophony acids or examinations of the complex isomerization and esterification processes. A new reaction scheme (figure 6) is proposed herein as a means of developing a suitable kinetic model including the colophony acids and products in accordance with the above reaction characteristics.

In this scheme, *k*_1_ and *k*_2_ are the rate constants for the isomerization of NA and PA to AA (A → C, B → C), *k*_3_ is the rate constant for the esterification of NA to MENA (A → D), *k*_4_ is the rate constant for the esterification of AA to MEA (C → E), *k*_5_ is the rate constant for the esterification of PA to MEPA (B → F) and *k*_6_ is the rate constant for the esterification of DA to MEDA (G → H).

The power function dynamics model is a rate equation that can be used for both homogeneous and heterogeneous reactions. In this case, methanol was present in excess and the product was dried in a vacuum oven, so the molar concentration was negligible [35]. The effects of internal and external diffusion were also eliminated by changing the catalyst particle size and agitator speed, so the reaction can be regarded as a quasi-first-order reaction. The relevant kinetic equations can be expressed as follows:

3.2 $$\begin{array}{r} \frac{\text{d}C_{A}}{\text{d}t}=-k_{1}C_{A}-k_{3}C_{A}, \end{array}$$

3.3 $$\begin{array}{r} \frac{\text{d}C_{B}}{\text{d}t}=-k_{2}C_{B}-k_{5}C_{B}, \end{array}$$

3.4 $$\begin{array}{r} \frac{\text{d}C_{C}}{\text{d}t}=k_{1}C_{A}+k_{2}C_{B}-k_{4}C_{C}, \end{array}$$

3.5 $$\begin{array}{r} \frac{\text{d}C_{D}}{\text{d}t}=k_{3}C_{A}, \end{array}$$

3.6 $$\begin{array}{r} \frac{\text{d}C_{E}}{\text{d}t}=k_{4}C_{C}, \end{array}$$

3.7 $$\begin{array}{r} \frac{\text{d}C_{F}}{\text{d}t}=k_{5}C_{B}, \end{array}$$

3.8 $$\begin{array}{r} \frac{\text{d}C_{G}}{\text{d}t}=-k_{6}C_{G} \end{array}$$

3.9 $$\begin{array}{r} \;\;\text{and}\;\;\;\;\frac{\text{d}C_{H}}{\text{d}t}=k_{6}C_{G}, \end{array}$$

where *C*_A_, *C*_B_, *C*_C_, *C*_D_, *C*_E_, *C*_F_, *C*_G_, and *C*_H_ represent the concentration of each component and *t* represents the sampling time. The concentration of each component can be calculated based on the mass fractions obtained from HPLC analysis and employing the following equation:

3.10 $$C_{i}=\frac{w_{i}\times W_{0}\times d}{M_{i}\times W}\times 1000,$$

where *C* is the concentration of a component, *w* is the mass fraction, *W*_0_ is the combined mass of colophony and methanol, *d* is the density of the liquid product, *W* is the product mass, *M* is the molecular weight of the component and *i* is the component number.

#### 3.5.2. Modelling results and analysis

**Figure 7.**
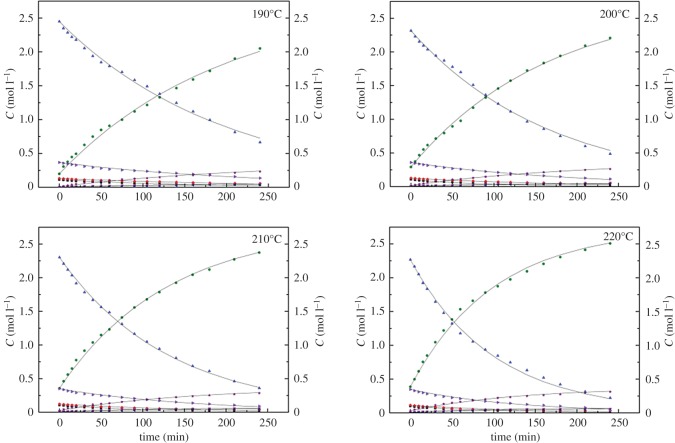
A comparison of the experimental (data point) and calculated values (line) of colophony acids and esters at various temperatures over the ZnO/SFCCR catalyst in conjunction with sub-CO_2_. Reaction conditions: a CO_2_ pressure of 3.5 MPa, a catalyst : colophony mass ratio of 1.0% and a methanol : colophony molar ratio of 1.5 : 1. Legend: (▪) NA, (⧫) PA, (▴) AA, (▾) MENA, (•) MEA, (◂) MEPA, (▸) DA, (★) MEDA.

The kinetics of the colophony methyl esterification process were studied under the optimum conditions, consisting of a temperature range of 190–220°C, a reaction time of 4 h, a CO_2_ pressure of 4 MPa, a catalyst : colophony mass ratio of 1.0%, a methanol : colophony molar ratio of 1.5 : 1 and a stirring rate of 400 r.p.m. (to reduce mass transfer effects). The resulting concentrations and variations of different colophony acids and methyl esters are summarized in figure 7. The rate constants were calculated using the Levenberg–Marquardt nonlinear least-squares method and employing the Matlab software package, and the activation energies (*Ea*) and frequency factors (*k*_0_) were estimated from the Arrhenius equation (equation (3.11)). The results are shown in table 3.

3.11 $$K=k_{0}\exp\left(-\frac{Ea}{\text{RT}}\right).$$

The experiments were performed under 3.5 Mpa of CO_2_ for 4 h, the catalyst : colophony mass ratio is 1.0% and the methanol : colophony molar ratio is 1.5 : 1.

**Table 3.** Kinetic parameters for the isomerization and esterification of colophony with methanol. The experiments were performed under 3.5 Mpa of CO_2_ for 4 h, the catalyst : colophony mass ratio is 1.0% and the methanol : colophony molar ratio is 1.5 : 1.

	temperature (°C)					
kinetic constant	190	200	210	220	*k*_0_ (min^−1^)	*E*_a_ (kJ mol^−1^)
*k*_1_(min^−1^)	0.0010 ± 0.0002	0.0018 ± 0.0002	0.0026 ± 0.0001	0.0039 ± 0.0005	1.1973 × 10^9^	107.09
*k*_2_(min^−1^)	0.0008 ± 0.0001	0.0013 ± 0.0003	0.0023 ± 0.0002	0.0032 ± 0.0003	2.7202 × 10^9^	113.95
*k*_3_(min^−1^)	0.0023 ± 0.0003	0.0036 ± 0.0004	0.0051 ± 0.0007	0.0066 ± 0.0007	8.6965 × 10^4^	68.99
*k*_4_(min^−1^)	0.0033 ± 0.0006	0.0057 ± 0.0007	0.0080 ± 0.0012	0.0103 ± 0.0015	1.9607 × 10^3^	49.85
*k*_5_(min^−1^)	0.0012 ± 0.0001	0.0021 ± 0.0003	0.0029 ± 0.0004	0.0041 ± 0.0008	7.0364 × 10^4^	75.43
*k*_6_(min^−1^)	0.0027 ± 0.0002	0.0046 ± 0.0005	0.0060 ± 0.0003	0.0077 ± 0.0009	6.5336 × 10^4^	59.20

This model includes the isomerization of NA and PA to AA and esterification to the methyl esters over the ZnO/SFCCR with sub-CO_2_. Table 3 demonstrates that the reaction rate constants were reduced in the order of *k*_4_*> k*_6* *_*> k*_3* *_*> k*_5_ on going from 190 to 220°C, indicating that the primary reaction during esterification was the generation of MEA. The changes in the concentration of each component demonstrate the same trend (figure 7). In the case of the isomerization, *k*_1_ is greater than *k*_2_, with values of 0.0039 and 0.0032 min^−1^, respectively, at 220°C. In addition, *k*_3 _*> k*_1_ and *k*_5_* > k*_2_, indicating that ZnO/SFCCR with sub-CO_2_ promotes esterification over isomerization, especially the esterification of NA.

In the case of a true kinetically controlled reaction, *Ea* should be in the range of 15–400 kJ mol^−1^, and the data are within these limits [36]. For the methyl esterification reaction, *Ea*_5 _> *Ea*_3 _> *Ea*_6 _> *Ea*_4_, suggesting that esterification of MEPA is more sensitive to temperature. In addition, the *Ea* values for NA and PA esterification are lower than those for isomerization, which is consistent with the experimental results and suggests that esterification becomes less favourable among the competing reactions at higher temperatures.

#### 3.5.3. Verification of the kinetic model

A good fit of the kinetic parameters can be used to illustrate the accuracy of the mathematical models. As seen in figure 7, the calculated concentrations obtained from the model are in good agreement with the experimental data in the temperature range of 190–220°C. An *F*-test was performed at a confidence interval of 95% to assess the accuracy of the model results and reasonable confidence intervals for the estimated parameters can be seen in table 3, and the results of statistical tests are shown in table 4. It is obvious that each of the correlation coefficients (*ρ*^2^) is larger than 0.90 and that the *F*-values are greater than the *F_t_*-value (*F*_0.05_ (5,13) = 3.03) multiplied by 10, demonstrating that the kinetic model is significant at the 95% confidence level.

**Table 4.** Results of statistical tests of the proposed model for esterification of colophony acids with methanol.

temperature (°C)	*ρ*^2^	*Q*	*F*
190	0.982	0.0375	900.189
200	0.987	0.0533	1184.623
210	0.996	0.107	1351.147
220	0.973	0.0829	1231.831

## 4. Conclusion

SFCCR, a solid waste generated by the petroleum refining process, can be used to synthesize a new ZnO/SFCCR heterogeneous catalyst for the preparation of colophony esters. With the addition of sub-CO_2_ as an auxiliary catalyst, excellent results were obtained during the esterification of colophony with methanol. Apart from providing auxiliary acid catalysis (a pH range of 3.54–3.91), the sub-CO_2_ acted to improve the conversion rate by removing water generated during the reaction, reducing the viscosity of the system, promoting gas–liquid mass transfer and increasing the lifespan of ZnO/SFCCR. The optimum reaction conditions were as follows: a CO_2_ pressure of 3.5 MPa, a catalyst : colophony mass ratio of 1.0%, a methanol : colophony molar ratio of 1.5 : 1, a reaction temperature of 220°C and a reaction time of 5 h. These conditions gave a high conversion rate of 97.01%. A novel experimental set-up with a new injection method and a special sampling device was designed to study the kinetics during the production of CMEs, and a new reaction pathway was proposed. The kinetic parameters were estimated using the Levenberg–Marquardt nonlinear least-squares method in conjunction with Matlab programming. The results indicated that MEA was formed more readily than other products, and that the methyl esterification of colophony acids proceeded preferentially over isomerization. The kinetic parameters of each reaction were in agreement with the experimental data based on *F*-tests at the 95% confidence level. This study suggests that similar catalysts are likely to promote the preparation of other colophony esters.

## Supplementary Material

rsos-172124 supplementary materials

## Data accessibility

The datasets supporting this article have been uploaded as part of the electronic supplementary material.
