# Supplementary material for: Catalytic methyl esterification of colophony over ZnO/SFCCR with subcritical CO2: catalytic performance, reaction pathway and kinetics
Source: R Soc Open Sci. 2018 May 2;5(5):172124. doi: 10.1098/rsos.172124 (PMC5990756; doi:10.1098/rsos.172124)
Supplement: rsos-172124 supplementary materials [file rsos172124supp1.docx]

**Royal Society Open Science**

**Supplementary information**

**Catalytic methyl esterification of colophony over ZnO/SFCCR with subcritical CO_2_: catalytic performance, reaction pathway and kinetics**

Xubin Wang , Linlin Wang ^a^***, Xiaopeng Chen ^a^**,* Dan Zhou ^a^, Han Xiao ^a,b^, Xiaojie Wei ^a^, Jiezhen Liang^a^

**Figure S1.** FTIR spectrum of SFCCR (a) and ZnO/SFCCR (b).





**Figure S2.** Py-IR spectrum of SFCCR (a) , ZnO (b) and ZnO/SFCCR (C).


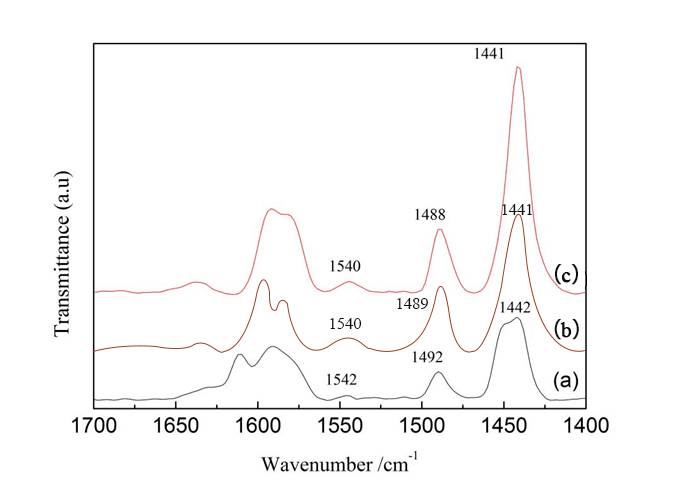


**Table S1**. Results from the elemental analysis of the ZnO/SFCCR catalyst using SEM–EDS (wt %).

| element | O | Si | Al | C | Ca | Zn | Cl | Fe |
| --- | --- | --- | --- | --- | --- | --- | --- | --- |
| Content (%) | 35.22 | 11.43 | 16.42 | 8.07 | 0.58 | 17.94 | 9.28 | 1.06 |

**Table S2.** BET surface area, micropore volume and pore diameter.

| Sample | BET surface areas (m^2^g^-1^) | Micropore volume (ml/g) | Pore diameter (×10^-9^m) |
| --- | --- | --- | --- |
| SFCCR | 65.78 | 0.11 | 7.75 |
| SFCCR (pretreated) | 70.13 | 0.14 | 8.14 |
| ZnO/SFCCR | 99.98 | 0.13 | 10.15 |

**Table S3.** Results of ICP-AES compositional analysis of ZnO and ZnO/SFCCR.

| Sample | Al_2_O_3_ （%） | SiO_2_（%） | ZnO （%） |
| --- | --- | --- | --- |
| SFCCR | 49.27 | 37.64 | 0 |
| ZnO/SFCCR | 36.51 | 27.93 | 20.85 |

**Table S4.** Results of replicate catalyst stability trials.

| Times catalyst recycled | 1 | 2 | 3 | 4 | 5 | 6 |
| --- | --- | --- | --- | --- | --- | --- |
| Conversions (SFCCR with N_2_,%) | 86.33 | 85.79 | 84.91 | 82.63 | 76.83 | 63.47 |
| Conversions (SFCCR with CO_2_,%) | 97.01 | 96.88 | 96.03 | 95.11 | 93.89 | 93.54 |

**Table S5.** Effects of the CO_2_ pressure on the colophony esterification. Reaction conditions: catalyst amount = 1.0 wt%, methanol:colophony molar ratio = 1.5:1, temperature = 220 ℃, time = 4 h, agitator speed = 600 rpm.

| Pco_2_ (M Pa) | Conversion（%） | Viscosity（m Pa·s） |
| --- | --- | --- |
| 0 | 83.28 | 4910 |
| 2 | 90.25 | 4230 |
| 3 | 92.69 | 4070 |
| 3.5 | 94.23 | 3880 |
| 4 | 94.08 | 3920 |
| 6 | 94.26 | 3920 |

**Table S6.** Effects of the catalyst amount on the colophony esterification. Reaction conditions: CO_2_ pressure = 3.5 MPa, methanol:colophony molar ratio = 1.5:1, temperature = 220 ℃, time = 4 h, agitator speed = 600 rpm.

| Catalyst dosage (%) | Conversion（%） | Viscosity（m Pa·s） |
| --- | --- | --- |
| 0 | 59.42 | 13790 |
| 0.5 | 86.87 | 4630 |
| 1 | 94.23 | 3880 |
| 1.5 | 93.88 | 3910 |
| 2.5 | 90.99 | 4020 |

**Table S7.** Effects of the methanol:colophony molar ratio on the colophony esterification. Reaction conditions: CO_2_ pressure = 3.5 MPa, catalyst amount = 1.0 wt%, temperature = 220 ℃, time = 4 h, agitator speed = 600 rpm.

| molar ratio of methanol to colophony | Conversion（%） | Viscosity（m Pa·s） |
| --- | --- | --- |
| 1.25 | 88.84 | 4630 |
| 1.5 | 94.23 | 3880 |
| 1.75 | 90.67 | 3540 |
| 2 | 86.64 | 3110 |

**Table S8.** Effects of the reaction temperature on the colophony esterification. Reaction conditions: CO_2_ pressure = 3.5 MPa, catalyst amount = 1.0 wt%, methanol:colophony molar ratio = 1.5:1, time = 4 h, agitator speed = 600 rpm.

| Temperature (℃) | Conversion（%） | Viscosity（m Pa·s） |
| --- | --- | --- |
| 200 | 84.43 | 4880 |
| 210 | 89.32 | 4170 |
| 220 | 94.23 | 3880 |
| 230 | 93.99 | 3390 |
| 240 | 91.60 | 2870 |

**Table S9.** Effects of the reaction time on the colophony esterification. Reaction conditions: CO_2_ pressure = 3.5 MPa, catalyst amount = 1.0 wt%, methanol:colophony molar ratio = 1.5:1, temperature = 220 ℃, agitator speed = 600 rpm.

| Time (h) | Conversion（%） | Viscosity（m Pa·s） |
| --- | --- | --- |
| 2 | 77.45 | 10360 |
| 3 | 86.63 | 5130 |
| 4 | 94.23 | 3880 |
| 4.5 | 95.91 | 3820 |
| 5 | 96.92 | 3810 |
| 6 | 97 | 3810 |

**Table S10.** Kinetic datas of concentrations and variations of different colophony acids and methyl esters. Reaction conditions: a CO_2_ pressure of 3.5 MPa, a catalyst:colophony mass ratio of 1.0% and a methanol:colophony molar ratio of 1.5:1, a reaction temperature of 190 °C.

| t/min | PA (mol L^-1^) | AA (mol L^-1^) | DA  (mol L^-1^) | NA  (mol L^-1^) | MEPA  (mol L^-1^) | MEA  (mol L^-1^) | MEDA  (mol L^-1^) | MENA  (mol L^-1^) |
| --- | --- | --- | --- | --- | --- | --- | --- | --- |
| 0 | 0.1318 | 2.4484 | 0.3667 | 0.1123 | 0.0029 | 0.1994 | 0.0086 | 0.0032 |
| 5 | 0.1266 | 2.3485 | 0.3553 | 0.1054 | 0.0042 | 0.3067 | 0.0172 | 0.0066 |
| 10 | 0.1228 | 2.2857 | 0.3462 | 0.0999 | 0.0045 | 0.3762 | 0.0273 | 0.0089 |
| 15 | 0.1190 | 2.2207 | 0.3371 | 0.0943 | 0.0053 | 0.4471 | 0.0392 | 0.0116 |
| 20 | 0.1160 | 2.1781 | 0.3278 | 0.0901 | 0.0058 | 0.4945 | 0.0489 | 0.0135 |
| 30 | 0.1105 | 2.0540 | 0.3089 | 0.0831 | 0.0072 | 0.6264 | 0.0660 | 0.0168 |
| 40 | 0.1059 | 1.9382 | 0.2945 | 0.0776 | 0.0084 | 0.7488 | 0.0790 | 0.0191 |
| 50 | 0.1017 | 1.8454 | 0.2801 | 0.0731 | 0.0097 | 0.8471 | 0.0944 | 0.0210 |
| 60 | 0.0979 | 1.7894 | 0.2665 | 0.0691 | 0.0113 | 0.9074 | 0.1073 | 0.0229 |
| 75 | 0.0927 | 1.7029 | 0.2561 | 0.0639 | 0.0140 | 0.9982 | 0.1180 | 0.0263 |
| 90 | 0.0887 | 1.5832 | 0.2467 | 0.0597 | 0.0162 | 1.1212 | 0.1268 | 0.0290 |
| 105 | 0.0853 | 1.4905 | 0.2381 | 0.0563 | 0.0182 | 1.2165 | 0.1354 | 0.0312 |
| 120 | 0.0829 | 1.3795 | 0.2254 | 0.0533 | 0.0195 | 1.3296 | 0.1480 | 0.0332 |
| 140 | 0.0758 | 1.2458 | 0.2095 | 0.0488 | 0.0257 | 1.4650 | 0.1642 | 0.0369 |
| 160 | 0.0685 | 1.1194 | 0.1943 | 0.0443 | 0.0323 | 1.5928 | 0.1792 | 0.0407 |
| 180 | 0.0646 | 0.9939 | 0.1798 | 0.0401 | 0.0357 | 1.7194 | 0.1923 | 0.0443 |
| 210 | 0.0593 | 0.8144 | 0.1576 | 0.0351 | 0.0406 | 1.8999 | 0.2140 | 0.0487 |
| 240 | 0.0570 | 0.6643 | 0.1359 | 0.0305 | 0.0425 | 2.0510 | 0.2331 | 0.0526 |

**Table S11.** Kinetic datas of concentrations and variations of different colophony acids and methyl esters. Reaction conditions: a CO_2_ pressure of 3.5 MPa, a catalyst:colophony mass ratio of 1.0% and a methanol:colophony molar ratio of 1.5:1, a reaction temperature of 200 °C.

| t/min | PA (mol L^-1^) | AA (mol L^-1^) | DA  (mol L^-1^) | NA  (mol L^-1^) | MEPA  (mol L^-1^) | MEA  (mol L^-1^) | MEDA  (mol L^-1^) | MENA  (mol L^-1^) |
| --- | --- | --- | --- | --- | --- | --- | --- | --- |
| 0 | 0.1270 | 2.3150 | 0.3580 | 0.1054 | 0.0041 | 0.2918 | 0.0154 | 0.0063 |
| 5 | 0.1217 | 2.2288 | 0.3461 | 0.0984 | 0.0046 | 0.3725 | 0.0271 | 0.0088 |
| 10 | 0.1166 | 2.1505 | 0.3354 | 0.0907 | 0.0055 | 0.4668 | 0.0380 | 0.0126 |
| 15 | 0.1120 | 2.0922 | 0.3259 | 0.0857 | 0.0066 | 0.5520 | 0.0472 | 0.0142 |
| 20 | 0.1081 | 2.0346 | 0.3147 | 0.0811 | 0.0074 | 0.6165 | 0.0592 | 0.0157 |
| 30 | 0.1029 | 1.9440 | 0.2989 | 0.0770 | 0.0081 | 0.7145 | 0.0749 | 0.0174 |
| 40 | 0.0981 | 1.8703 | 0.2835 | 0.0707 | 0.0092 | 0.7956 | 0.0900 | 0.0200 |
| 50 | 0.0933 | 1.7781 | 0.2688 | 0.0645 | 0.0109 | 0.8942 | 0.1048 | 0.0233 |
| 60 | 0.0885 | 1.6993 | 0.2602 | 0.0592 | 0.0132 | 0.9775 | 0.1132 | 0.0262 |
| 75 | 0.0832 | 1.5088 | 0.2433 | 0.0536 | 0.0166 | 1.1718 | 0.1299 | 0.0299 |
| 90 | 0.0785 | 1.3599 | 0.2251 | 0.0486 | 0.0198 | 1.3238 | 0.1465 | 0.0333 |
| 105 | 0.0714 | 1.2304 | 0.2113 | 0.0439 | 0.0258 | 1.4556 | 0.1616 | 0.0368 |
| 120 | 0.0647 | 1.1145 | 0.1984 | 0.0399 | 0.0316 | 1.5733 | 0.1768 | 0.0401 |
| 140 | 0.0598 | 0.9647 | 0.1795 | 0.0359 | 0.0358 | 1.7245 | 0.1962 | 0.0434 |
| 160 | 0.0557 | 0.8552 | 0.1625 | 0.0324 | 0.0394 | 1.8350 | 0.2141 | 0.0464 |
| 180 | 0.0538 | 0.7499 | 0.1483 | 0.0295 | 0.0410 | 1.9410 | 0.2293 | 0.0489 |
| 210 | 0.0516 | 0.5998 | 0.1273 | 0.0248 | 0.0430 | 2.0916 | 0.2482 | 0.0533 |
| 240 | 0.0493 | 0.4852 | 0.1129 | 0.0206 | 0.0451 | 2.2067 | 0.2621 | 0.0570 |

**Table S12.** Kinetic datas of concentrations and variations of different colophony acids and methyl esters. Reaction conditions: a CO_2_ pressure of 3.5 MPa, a catalyst:colophony mass ratio of 1.0% and a methanol:colophony molar ratio of 1.5:1, a reaction temperature of 210 °C.

| t/min | PA (mol L^-1^) | AA (mol L^-1^) | DA  (mol L^-1^) | NA  (mol L^-1^) | MEPA  (mol L^-1^) | MEA  (mol L^-1^) | MEDA  (mol L^-1^) | MENA  (mol L^-1^) |
| --- | --- | --- | --- | --- | --- | --- | --- | --- |
| 0 | 0.1227 | 2.3015 | 0.3499 | 0.1039 | 0.0046 | 0.3566 | 0.0259 | 0.0086 |
| 5 | 0.1167 | 2.2057 | 0.3358 | 0.0995 | 0.0053 | 0.4623 | 0.0441 | 0.0124 |
| 10 | 0.1108 | 2.1175 | 0.3181 | 0.0888 | 0.0066 | 0.5592 | 0.0584 | 0.0150 |
| 15 | 0.1053 | 2.0350 | 0.3071 | 0.0831 | 0.0078 | 0.6495 | 0.0696 | 0.0172 |
| 20 | 0.1004 | 1.9161 | 0.2894 | 0.0784 | 0.0088 | 0.775 | 0.0864 | 0.0192 |
| 30 | 0.0935 | 1.7829 | 0.2729 | 0.0738 | 0.0105 | 0.9156 | 0.1028 | 0.0216 |
| 40 | 0.0882 | 1.6681 | 0.2602 | 0.0660 | 0.0125 | 1.0375 | 0.1156 | 0.0256 |
| 50 | 0.0829 | 1.5625 | 0.2509 | 0.0600 | 0.0151 | 1.1489 | 0.1251 | 0.0285 |
| 60 | 0.0784 | 1.4842 | 0.2418 | 0.0548 | 0.0173 | 1.2321 | 0.1341 | 0.0311 |
| 75 | 0.0701 | 1.3117 | 0.2218 | 0.0487 | 0.0236 | 1.4088 | 0.1537 | 0.0350 |
| 90 | 0.0642 | 1.1659 | 0.2049 | 0.0434 | 0.0279 | 1.5579 | 0.1709 | 0.0386 |
| 105 | 0.0574 | 1.0487 | 0.1877 | 0.0386 | 0.0337 | 1.6778 | 0.1884 | 0.0420 |
| 120 | 0.0534 | 0.9425 | 0.1731 | 0.0344 | 0.037 | 1.7859 | 0.2041 | 0.0450 |
| 140 | 0.0502 | 0.8053 | 0.1523 | 0.0295 | 0.0398 | 1.9245 | 0.2238 | 0.0489 |
| 160 | 0.0474 | 0.6866 | 0.1375 | 0.0256 | 0.0423 | 2.0442 | 0.2385 | 0.0521 |
| 180 | 0.0451 | 0.6109 | 0.1235 | 0.0222 | 0.0444 | 2.1205 | 0.2526 | 0.0551 |
| 210 | 0.0435 | 0.4602 | 0.1066 | 0.0193 | 0.0459 | 2.2716 | 0.2683 | 0.0577 |
| 240 | 0.0425 | 0.3580 | 0.0908 | 0.0173 | 0.0468 | 2.3741 | 0.2839 | 0.0589 |

**Table S13.** Kinetic datas of concentrations and variations of different colophony acids and methyl esters. Reaction conditions: a CO_2_ pressure of 3.5 MPa, a catalyst:colophony mass ratio of 1.0% and a methanol:colophony molar ratio of 1.5:1, a reaction temperature of 220 °C.

| t/min | PA (mol L^-1^) | AA (mol L^-1^) | DA  (mol L^-1^) | NA  (mol L^-1^) | MEPA  (mol L^-1^) | MEA  (mol L^-1^) | MEDA  (mol L^-1^) | MENA  (mol L^-1^) |
| --- | --- | --- | --- | --- | --- | --- | --- | --- |
| 0 | 0.1152 | 2.2668 | 0.3471 | 0.0998 | 0.0049 | 0.3868 | 0.0287 | 0.0093 |
| 5 | 0.1087 | 2.1651 | 0.3269 | 0.0910 | 0.0059 | 0.4985 | 0.0493 | 0.0136 |
| 10 | 0.1026 | 2.0521 | 0.3103 | 0.0840 | 0.0071 | 0.6154 | 0.0660 | 0.0167 |
| 15 | 0.0968 | 1.9215 | 0.2975 | 0.0783 | 0.0083 | 0.7539 | 0.0794 | 0.0191 |
| 20 | 0.0915 | 1.8336 | 0.2806 | 0.0737 | 0.0095 | 0.8487 | 0.0952 | 0.0209 |
| 30 | 0.0835 | 1.6441 | 0.2593 | 0.0665 | 0.0123 | 1.0459 | 0.1166 | 0.0256 |
| 40 | 0.0765 | 1.4785 | 0.2413 | 0.0573 | 0.0163 | 1.2196 | 0.1333 | 0.0307 |
| 50 | 0.0689 | 1.3221 | 0.2255 | 0.0503 | 0.0206 | 1.3827 | 0.1497 | 0.0343 |
| 60 | 0.0602 | 1.1763 | 0.2109 | 0.0444 | 0.0267 | 1.5339 | 0.1659 | 0.0374 |
| 75 | 0.0526 | 1.0551 | 0.1886 | 0.0378 | 0.0320 | 1.6597 | 0.1858 | 0.0417 |
| 90 | 0.0467 | 0.9376 | 0.1728 | 0.0330 | 0.0364 | 1.7805 | 0.2023 | 0.0447 |
| 105 | 0.0416 | 0.8460 | 0.1551 | 0.0287 | 0.0404 | 1.8747 | 0.2205 | 0.0475 |
| 120 | 0.0382 | 0.7465 | 0.1432 | 0.0244 | 0.0430 | 1.9763 | 0.2330 | 0.0505 |
| 140 | 0.0362 | 0.6299 | 0.1273 | 0.0196 | 0.0445 | 2.0944 | 0.2484 | 0.0543 |
| 160 | 0.0349 | 0.5204 | 0.1138 | 0.0159 | 0.0455 | 2.2049 | 0.2613 | 0.0573 |
| 180 | 0.0334 | 0.4209 | 0.1017 | 0.0136 | 0.0468 | 2.3061 | 0.2739 | 0.0591 |
| 210 | 0.0325 | 0.3133 | 0.0794 | 0.0123 | 0.0476 | 2.4142 | 0.2962 | 0.0599 |
| 240 | 0.0319 | 0.2206 | 0.0352 | 0.0117 | 0.0480 | 2.5072 | 0.3119 | 0.0602 |
